# Correlation between healthy sleep score and risk of cardia-cerebrovascular disease among people with type 2 diabetes: a prospective cohort study

**DOI:** 10.3389/fcvm.2025.1640125

**Published:** 2026-01-08

**Authors:** Dasen Sang, Jingxiang Wang, Yao Zhang, Shouling Wu, Jie Tao, Wei Geng

**Affiliations:** 1 Department of Cardiology, Baoding NO.1 Central Hospital, Baoding, Hebei, China; 2 Department of Cardiology, Kailuan General Hospital, Tangshan, Hebei, China

**Keywords:** cardia-cerebrovascular disease, sleep, healthy sleep score, type 2 diabetes, risk factor

## Abstract

**Background:**

In the general population, healthy sleep pattern is associated with lower risk of cardia-cerebrovascular diseases (CVD). However, despite a high prevalence of sleep disorders in people type 2 diabetes(T2D), no study has investigated the relationship between sleep patterns and the risk of CVD events in this particular subpopulation.

**Methods:**

We included 6,363 participants with T2D but free of prevalent CVD at baseline from Kailuan study, a HSS(range 0–5) combining five sleep patterns (sleep duration, snoring, insomnia, early sleep-wake patterns, and excessive daytime sleepiness) was calculated. Cox regression models were used to evaluate the hazard ratios (HRs) and 95% confidence intervals (CIs) of incident CVD.

**Results:**

During a median follow-up of 5.80 years, 790 participants developed first CVD event (12.42%). In multivariate Cox analysis, the risk of CVD decreased by 11% (HR, 0.89; 95% CI 0.83–0.96) per one-point increment in the HSS. Compared to those with a sleep score of 0–1, participants with a score of 4 and 5 had a 26%(HR, 0.73; 95% CI 0.55–0.99) and 43%(HR, 0.58; 95% CI 0.35–0.93) reduced risk of CVD, respectively.

**Conclusion:**

Higher HSS are associated with a lower risk of CVD events in the community people with T2D.

## 1 Introduction

Sleep is a fundamental component of human biology, influencing nearly every physiological system (1, 2).Epidemiological studies have established that inappropriate sleep duration—including longer and shorter durations than the optimal duration—is a risk factor for the onset and progression of cardia-cerebrovascular disease (CVD) (3–5). Research has also shown that changes in sleep duration in real-life settings can affect cardiovascular risk factors (6). Recognizing this, the American Heart Association (AHA) added sleep duration to its “Life's Essential 8” in 2022, joining seven other key behaviors and health factors linked to heart health (7, 8). This highlights the growing need to explore how sleep affects the risk of cardiovascular disease.

Notably, healthy sleep is a multidimensional construct. Sleep duration reflects only one aspect of sleep behavior, whereas other essential dimensions include timing, regularity, efficiency, satisfaction, and its impact on daytime alertness. Most previous studies examining the association between sleep and CVD have focused on individual sleep behaviors. In order to better assess overall sleep patterns and their effects on CVD, a novel multidimensional sleep evaluation method—the healthy sleep score (HSS)—has been introduced. HSS encompasses sleep duration, chronotype, insomnia, sleep apnea or snoring, and daytime sleepiness. Only several relevant studies, to the best of the author's knowledge, have been conducted in the general population, all of which demonstrated that higher HSS scores are associated with a lower risk of CVD, including coronary heart disease and stroke (9–12), suggesting that poor sleep health is a significant risk factor for CVD.

Diabetes is an independent risk factor for CVD, and individuals with diabetes are at significantly greater risk (2–4 times) of developing CVD than those without diabetes (13). Using sleep health assessment in people with diabetes, in conjunction with traditional risk factors, may facilitate the identification of high-risk individuals and the subsequent implementation of targeted interventions to prevent CVD, which is of critical importance. Research data indicate that both insufficient and excessive sleep duration are more prevalent among individuals with diabetes than in the nondiabetic population and that both are associated with an increased risk of CVD (14, 15). The long-term impact of poor sleep health on individuals with diabetes, particularly its potential influence on CVD risk, remains an important research topic that has been understudied and warrants further investigation. Therefore, data from the Kailuan study cohort was used in this study to evaluate the association between the HSS score and the risk of incident CVD, including coronary heart disease, stroke, and peripheral artery disease, in individuals with diabetes.

## 2 Methods

### 2.1 Study design and population

This study is a prospective cohort study based on the Kailuan Study. In 11 hospitals, including Kailuan General Hospital and its affiliated hospitals, biennial health examinations for active and retired employees of the Kailuan Group were conducted from June 2006 to October 2007 (baseline survey in 2006). The health examinations included face-to-face questionnaire interviews, laboratory tests, and physical examinations, with follow-ups that recorded CVD events and mortality. Individuals with type 2 diabetes who participated in the 2016 health examination (the sixth survey cycle) were selected as the study population. In addition to routine health assessments, a sleep questionnaire was administered. The diagnosis of diabetes was based on the 2010 American Diabetes Association (ADA) guidelines for the diagnosis and management of diabetes (16).

This study was conducted in accordance with the Declaration of Helsinki and was approved by the Ethics Committee of Kailuan General Hospital. All participants provided written informed consent for the study.

In the current analysis, we included 7,947 participants with type 2 diabetes who participated in the 2016 health examination and were free from CVD (including heart failure, atrial fibrillation, myocardial infarction, stroke and peripheral vascular disease) at baseline. After excluding those who had missing or implausible values of sleep duration (i.e., <4 or >12 h/day), a total of 6,363 participants with type 2 diabetes remained.

### 2.2 Collection of general clinical data and laboratory investigations

General clinical data, including age, sex, comorbidities, medication use, and family history of CVD, were obtained through face-to-face questionnaire interviews. The measurement methods and standardized procedures for height, weight, blood pressure, and relevant biochemical parameters have been detailed in previously published studies (17). Current smokers were defined as those who smoked at least one cigarette per day on average in the past year or who had quit smoking for less than one year. Higher education was defined as attaining a senior high school diploma or above. Physical activity was defined as engaging in exercise at least 3 times per week, with each session lasting ≥30 min. Body mass index (BMI) was calculated using the following formula: BMI = weight (kg)/height^2^ (m^2^). The estimated glomerular filtration rate (eGFR) was calculated using the CKD-EPI equation (18). Albuminuria, determined through urinary albumin-to-creatinine ratio(uACR), includes microalbuminuria (uACR 3–30 mg/mmol), and macroalbuminuria (uACR ≥ 30 mg/mmol) categories (19).

### 2.3 Assessment of the HSS

The HSS consists of five components: sleep duration of 7–8 h per day, insomnia, daytime sleepiness, snoring, and early sleep-wake patterns. Since the Morningness-Eveningness Questionnaire (MEQ) was not included in this study, as a substitute, “early sleep-wake patterns” was used. The total score ranged from 0 to 5 points. Owing to the small number of participants scoring 0, scores of 0 and 1 were combined into a single category (0–1 points). The scoring criteria for each component were as follows:

(1) Sleep Duration: Participants who reported sleeping 7–8 h per day were assigned 1 point, whereas those with <7 h or >8 h per day were assigned 0 points.
(2) Insomnia: Based on the Pittsburgh Sleep Quality Index (PSQI), participants who reported having trouble falling asleep, waking up at night, or waking up too early “never” or less than once a week—and did not use sleep medication—were given 1 point. Those who experienced these issues “1–2 times per week, 3 or more times per week,” or used sleep medication were given 0 points.
(3) Daytime Sleepiness: Based on the PSQI, participants who reported “never” or “<1 time/week” of experiencing excessive daytime sleepiness were assigned 1 point, whereas those reporting “1–2 times/week” or “≥3 times/week” were assigned 0 points.
(4) Snoring: Participants who reported “never” or “occasionally” snoring were assigned 1 point, whereas those who reported “frequent” snoring were assigned 0 points.
(5) Early Sleep-Wake Patterns: Participants who reported going to bed between 20:00 and 21:00 and waking up between 05:00 and 06:00 or going to bed between 21:00 and 22:00 and waking up between 06:00 and 07:00 were assigned 1 point. Others were assigned 0 points.

### 2.4 Outcomes

The primary outcome event in this study was the occurrence of CVD, including myocardial infarction, coronary artery stent implantation, heart failure, and atrial fibrillation, as well as peripheral vascular disease and stroke (including hemorrhagic and ischemic stroke). We defined outcomes according to the International Classification of Diseases edition10 (ICD-10): I21 for myocardial infarction, I22 for coronary artery stent implantation, I50 for heart failure, I48 for atrial fibrillation, I70 for peripheral vascular disease, I61 for hemorrhagic stroke, and I63 for ischemic stroke. The follow-up period began at the time of the sixth health examination, and the endpoint was defined as the occurrence of a CVD event or all-cause mortality. For participants who did not experience a CVD event or death, the last follow-up date was December 31, 2022. Each year trained medical personnel reviewed the disease diagnosis records from the Kailuan Group, its affiliated hospitals, and designated hospitals under the municipal medical insurance system to track the occurrence of endpoint events. All diagnoses were confirmed by professional physicians on the basis of hospital medical records.

### 2.5 Statistical analysis

Normally distributed continuous variables were expressed as the mean ± standard deviation $\left(\bar{x}\pm s\right)$, and comparisons between multiple groups were performed using one-way analysis of variance (ANOVA) followed by pairwise comparisons. Non-normally distributed continuous variables were presented as medians (Q1, Q3), and comparisons between groups were conducted using the Kruskal–Wallis rank sum test. Categorical variables were presented as frequencies and percentages (*n*, %), and group comparisons were performed using the chi-squared test. A standardized mean difference (SMD) was used to compare baseline differences between the other HSS groups and the the 0–1 score group. A SMD of 10% between the two groups was defined as the threshold indicating effective balance and comparability.

Kaplan–Meier curves were used to estimate the incidence rates of CVD in each group, and the log-rank test was applied to compare differences between groups. To assess how different HSS levels and each additional point affected the risk of developing CVD, a multivariate Cox proportional hazards model was used. Models were adjusted for age (continuous) and sex (women or men), family history of CVD (yes or no), higher education(yes or no), smoking (yes or no), physical activity (yes or no), BMI (continuous), heart rate (continuous), HbA1c (continuous), LDL-C (continuous), HDL-C (continuous), eGFR (continuous), uACR (continuous), hs-CRP (continuous), and medications affecting sleep (yes or no). E-values were applied to assess the robustness against unmeasured confounders.

Considering that people with diabetes often have other metabolic conditions, subgroup analyses were carried out for individuals with and without hypertension, high cholesterol, and Albuminuria. To examine the robustness of our findings, several sensitivity analyses were also performed. These included repeating the Cox regression after removing one sleep factor at a time, reanalyzing after excluding participants who used medications like lipid-lowering, blood pressure, diabetes, or antiplatelet drugs. Also, we used the Fine-Gray subdistribution hazard model to evaluate the association between HSS and CVDs in the presence of competing events, with death considered as a competing risk factor. To exclude reverse causation bias, we excluded participants who developed CVDs in the first 2 years of follow- up.

SAS version 9.4 was used for the analysis (SAS Institute, Cary, NC, USA). All statistical analyses were double-tailed, with statistical significance set at *P* < 0.05.

## 3 Results

### 3.1 Baseline characteristics of participants

Among the 6,363 participants with type 2 diabetes, 4,971 (78.1%) were male, and the mean (SD) age at baseline was 60.1 (10.1) years. The mean (SD) HbA1c and SBP in the population were 7.6 (±1.7) % and 146.6 (±20.5) mmHg, respectively. Among all participants, 5.6% had a poor HSS (score = 0 or 1), whereas 3.9% had an optimal HSS (score = 5).

The baseline characteristics according to HSS categories are shown in Table 1. Participants with higher HSS were more likely to be man, non-smokers, to have higher measurements of SBP, and higher prevalence of hypertension.

**Table 1:** Baseline characteristics of participants according to the HSS (*n* = 6,363).

Variables	Overall *N* = 6,363	HSS								
		0–1 score *N* = 359	2 score *N* = 954	SMD	3 score *N* = 2,268	SMD	4 score *N* = 2,536	SMD	5 score *N* = 246	SMD
Male *n* (%)	4,971 (78.1)	236 (65.7)	720 (75.5)	0.21	1,792 (79.0)	0.30	2,029 (80.0)	0.32	194 (78.8)	0.29
Age years	60.1 ± 10.1	60.2 ± 9.9	59.7 ± 9.5	0.04	60.4 ± 9.8	0.02	59.8 ± 10.4	0.03	62.9 ± 10.4	0.27
Higher education *n* (%)	1,469 (23.1)	79 (22.0)	241 (25.3)	0.07	517 (22.8)	0.03	588 (23.2)	0.02	44 (17.9)	0.13
SBP mmHg	146.6 ± 20.5	143.8 ± 19.7	145.7 ± 20.0	0.01	147.5 ± 20.4	0.12	146.3 ± 20.8	0.07	147.9 ± 20.3	0.16
DBP mmHg	82.7 ± 10.9	81.6 ± 11.1	82.9 ± 11.2	0.05	83.3 ± 10.8	0.12	82.4 ± 10.8	0.02	81.5 ± 11.3	0.04
Heart rate bpm	78.5 ± 12.6	77.1 ± 12.4	78.1 ± 12.6	0.10	78.5 ± 12.7	0.13	78.9 ± 12.7	0.16	78.5 ± 10.8	0.14
BMI kg/m^2^	25.8 ± 3.4	26.0 ± 3.9	25.9 ± 3.4	0.03	25.9 ± 3.3	0.03	25.7 ± 3.4	0.10	25.5 ± 3.1	0.15
HDL-C*mmol/L	1.4 (1.2–1.6)	1.4 (1.2–1.7)	1.4 (1.2–1.6)	0.02	1.4 (1.2–1.6)	0.04	1.4 (1.1–1.6)	0.03	1.5 (1.2–1.7)	0.09
LDL-C mmol/L	3.3 ± 0.9	3.2 ± 0.9	3.3 ± 1.0	0.11	3.3 ± 0.9	0.12	3.3 ± 0.9	0.15	3.2 ± 0.9	0.10
FBG mmol/L	9.1 ± 3.2	9.3 ± 3.2	9.2 ± 3.2	0.00	9.1 ± 3.1	0.06	9.1 ± 3.2	0.04	9.2 ± 3.4	0.01
HbA1c %	7.6 ± 1.7	7.7 ± 1.6	7.6 ± 1.7	0.02	7.6 ± 1.6	0.06	7.6 ± 1.7	0.07	7.7 ± 1.8	0.01
Hs-CRP mg/L*	1.4 (0.5–3.7)	1.3 (0.5–3.7)	1.5 (0.6–3.7)	0.06	1.4 (0.5–3.5)	0.07	1.5 (0.5–4.1)	0.07	1.5 (0.5–4.3)	0.10
eGFR mL/min/1.73 m^2^	94.5 ± 15.4	97.1 ± 15.8	96.1 ± 15.2	0.06	94.4 ± 14.8	0.17	94.0 ± 15.7	0.19	91.7 ± 17.4	0.32
Smoker *n* (%)	1,716 (27.6)	106 (30.2)	315 (33.8)	0.07	664 (29.9)	0.01	598 (23.9)	0.14	42 (17.3)	0.30
Physical activity *n* (%)	4,140 (65.1)	228 (63.5)	632 (66.3)	0.06	1,465 (64.6)	0.02	1,660 (65.5)	0.04	155 (63.0)	0.01
Albuminuria *n* (%)	2,149 (33.8)	133 (37.0)	334 (35.1)	0.01	771 (34.0)	0.03	812 (32.0)	0.02	99 (40.2)	0.02
Family history of CVD n(%)	93 (1.5)	6 (1.7)	20 (2.1)	0.03	40 (1.8)	0.01	25 (1.0)	0.06	2 (0.8)	0.08
Hypertension *n* (%)	3,177 (49.9)	151 (42.1)	432 (45.3)	0.07	1,174 (51.8)	0.19	1,289 (50.8)	0.18	131 (53.3)	0.22
Diabetes medication *n* (%)	3,136 (49.6)	230 (64.1)	527 (55.2)	0.18	1,124 (49.6)	0.29	1,133 (44.7)	0.39	122 (49.6)	0.29
Antihypertensive medication *n* (%)	1,091 (17.2)	79 (22.0)	178 (18.7)	0.14	396 (17.5)	0.15	395 (15.6)	0.20	43 (17.5)	0.19
Anti-platelet therapy *n* (%)	228 (3.6)	19 (5.3)	57 (5.9)	0.03	86 (3.8)	0.07	63 (2.5)	0.15	3 (1.2)	0.23
Lipid-lowering medications *n* (%)	91 (1.4)	11 (3.1)	21 (2.2)	0.05	32 (1.4)	0.11	25 (1.0)	0.15	2 (0.8)	0.16
Sleep-affecting medications *n* (%)	329(5.2)	58(16.2)	137(14.4)	0.03	99(4.4)	0.39	30(1.2)	0.55	5(2.0)	0.52

SBP, systolic blood pressure; DBP, diastolic blood pressure; BMI, body mass index; HDL-C, high-density lipoprotein cholesterol; LDL-C, low-density lipoprotein cholesterol; FBG, fasting blood glucose; hs-CRP, highly sensitive C-reactive protein; eGFR, estimated glomerular filtration rate; HbA1c, glycated hemoglobin A1c.

* Expressed in M(Q1–Q3).

### 3.2 Associations between sleep characteristics and CVD incidence

**Figure 1:**
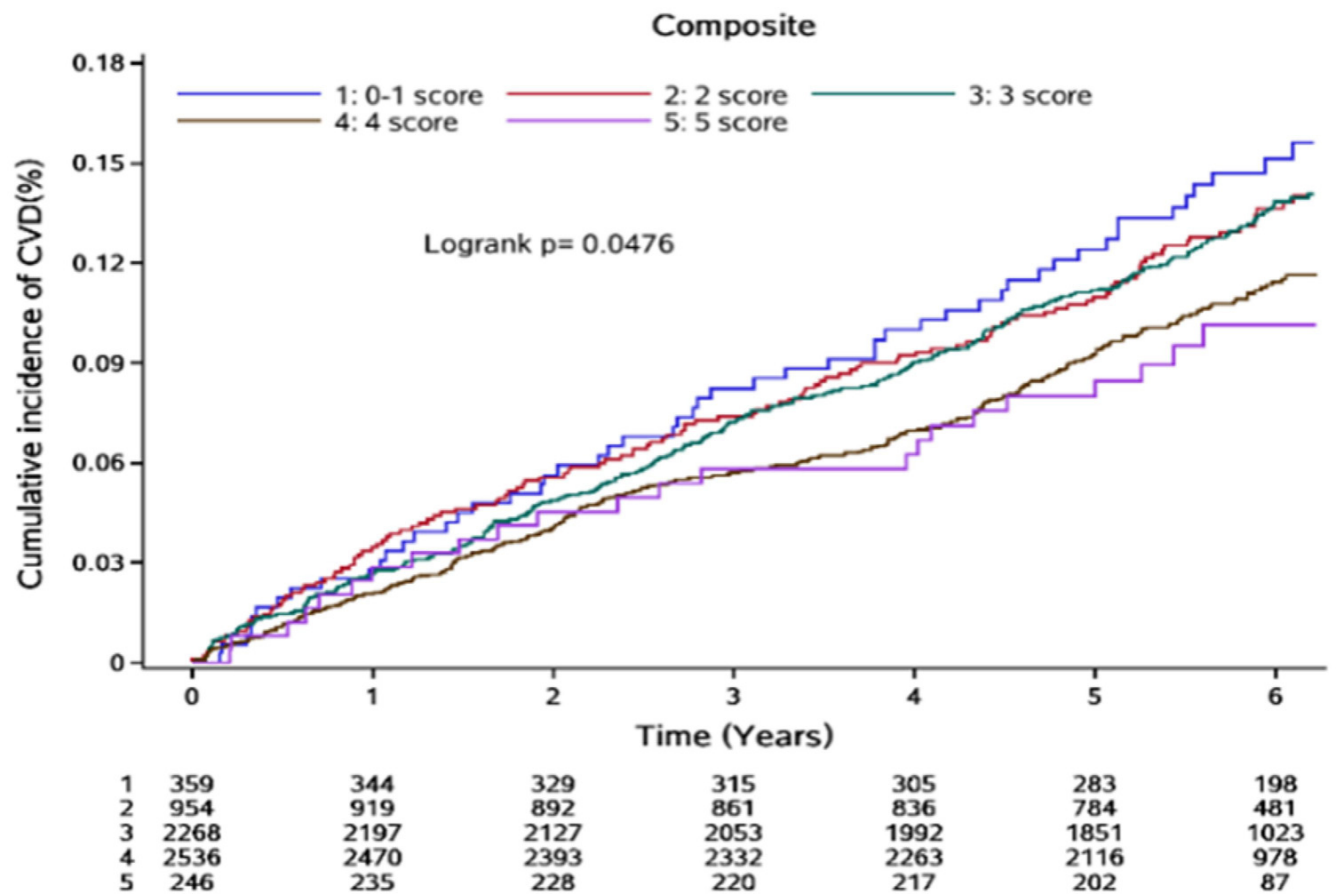
Cumulative incidence of CVD events by HSS scores.

Over a median follow-up of 5.80 years (range: 5.28–6.21 years), 790 CVD events were recorded, accounting for 12.42% of the study population. These included 130 cases of coronary heart disease (2.04%), 112 cases of heart failure (1.76%), 100 cases of atrial fibrillation (1.57%), 467 strokes (7.34%), and 57 cases of peripheral vascular disease (0.90%). Additionally, 80 participants experienced two or more CVD events. During the same period, 447 participants (7.02%) died. The cumulative incidence of CVD across the five HSS groups was 16.18%, 13.96%, 14.21%, 10.80%, and 8.86%, respectively. A log-rank test showed that these differences were statistically significant (*P* = 0.0476) (Figure 1).

### 3.3 Associations of sleep factors with CVD events

When the five sleep factors collapsed into binary categories of low risk vs. high risk (reference group), only free of insomnia and sleep 7–8 h/day were independently associated with incident CVD, with a 20% and 11% lower risk, respectively (Table 2).

**Table 2:** Multivariate Cox regression analysis of sleep factors effect on CVD events.

Sleep characteristics	Total CVD events			
	% of 6,363 participants	Model 1	Model 2	Model 3
Sleep 7–8 h/d	53.48	0.85 (0.74, 0.98)	0.86 (0.76, 0.99)	0.89 (0.76, 0.99)
Never/rarely insomnia	82.37	0.79 (0.69, 0.94)	0.80 (0.67, 0.95)	0.80 (0.66, 0.95)
No self-reported snoring	88.18	0.91 (0.74, 1.11)	0.83 (0.67, 1.02)	0.82 (0.66,1.01)
No frequent daytime sleepiness	88.61	0.87 (0.71, 1.07)	0.84 (0.68, 1.03)	0.87 (0.70, 1.07)
Early Sleep-Wake Patterns	8.2	1.01 (0.76, 1.41)	0.99 (0.78, 1.27)	0.99 (0.77, 1.27)

Model 1: Unadjusted.

Model 2: Adjusted for age and sex.

Model 3: Adjusted for age, sex, heart rate, BMI, LDL-C, HDL-C, eGFR, uACR, HbA1c, hs-CRP, hypertension status, family history of CVD, education, smoking status, physical activity and use of sleep-affecting medications. No significant interaction effect was noted between any of the sleep parameters and the odds of having a high risk for CVD (all *P* interactions > 0.05).

### 3.4 Associations of HSS scores with CVD events

When these five sleep factors were considered jointly using the HSS, the risk of CVD decreased significantly with increasing HSS (*P* for trend = 0.004). Compared to participants with scores of 0–1, those with scores of 4 and 5 had a 26% and 43% lower risk of CVD, respectively. When evaluated ordinarily, each additional HSS was associated with an 11% lower risk of CVD (HR for a one-point higher HSS = 0.89; 95% CI 0.83–0.96) (Table 3).

**Table 3:** Multivariate Cox regression analysis of HSS scores effect on CVD events.

HSS	Case/No.	Median follow-up (years)	Incidence rate (/1,000 PY)	Model 1	Model 2	Model 3*
0–1	54/359	6.09	27.90	Ref	Ref	Ref
2	129/954	6.02	24.78	0.89 (0.65, 1.22)	0.88 (0.64, 1.21)	0.89 (0.64, 1.22)
3	303/2,268	5.83	24.65	0.88 (0.66, 1.18)	0.86 (0.64, 1.14)	0.86 (0.64, 1.15)
4	281/2,536	5.73	20.61	0.74 (0.55, 0.99)	0.73 (0.54, 0.97)	0.74 (0.55, 0.99)
5	23/256	5.71	17.77	0.64 (0.39, 1.04)	0.57 (0.35, 0.92)	0.57 (0.35, 0.93)
Per + 1 score	-	-	-	0.90 (0.84, 0.97)	0.89 (0.83, 0.96)	0.89 (0.83, 0.96)
*P* trend	-	-	-	0.005	0.002	0.004

The data presented are HRs (95% CIs). Model 1: unadjusted. Model 2: adjusted for age and sex. Model 3: further adjusted for family history of CVD, education, smoking, physical activity, BMI, heart rate, HbA1c, LDL-C, HDL-C, eGFR, uACR, hs-CRP, and medications affecting sleep in Model 2. PY, person-years.

* *E* value = 1.50; lower limit of the CI(LL) = 1.25.

Further, it was noted that with an increase in the HSS score, the risk of both cardiovascular diseases and stroke decreased, even though there was no statistically significant association between the HSS score and stroke risk (Supplementary Table S1). When evaluated ordinally, each additional HSS was associated with a 12% lower risk of cardiovascular diseases (HR = 0.88; 95% CI 0.79–0.99) and an 8% lower risk of stroke (HR = 0.92; 95% CI 0.83–1.02). Cardiovascular diseases include myocardial infarction, coronary artery stenting, heart failure, atrial fibrillation, and peripheral vascular disease. Stroke includes both hemorrhagic and ischemic stroke.

### 3.5 Multivariate Cox regression subgroup analysis of HSS effect on new-onset CVD events

**Figure 2:**
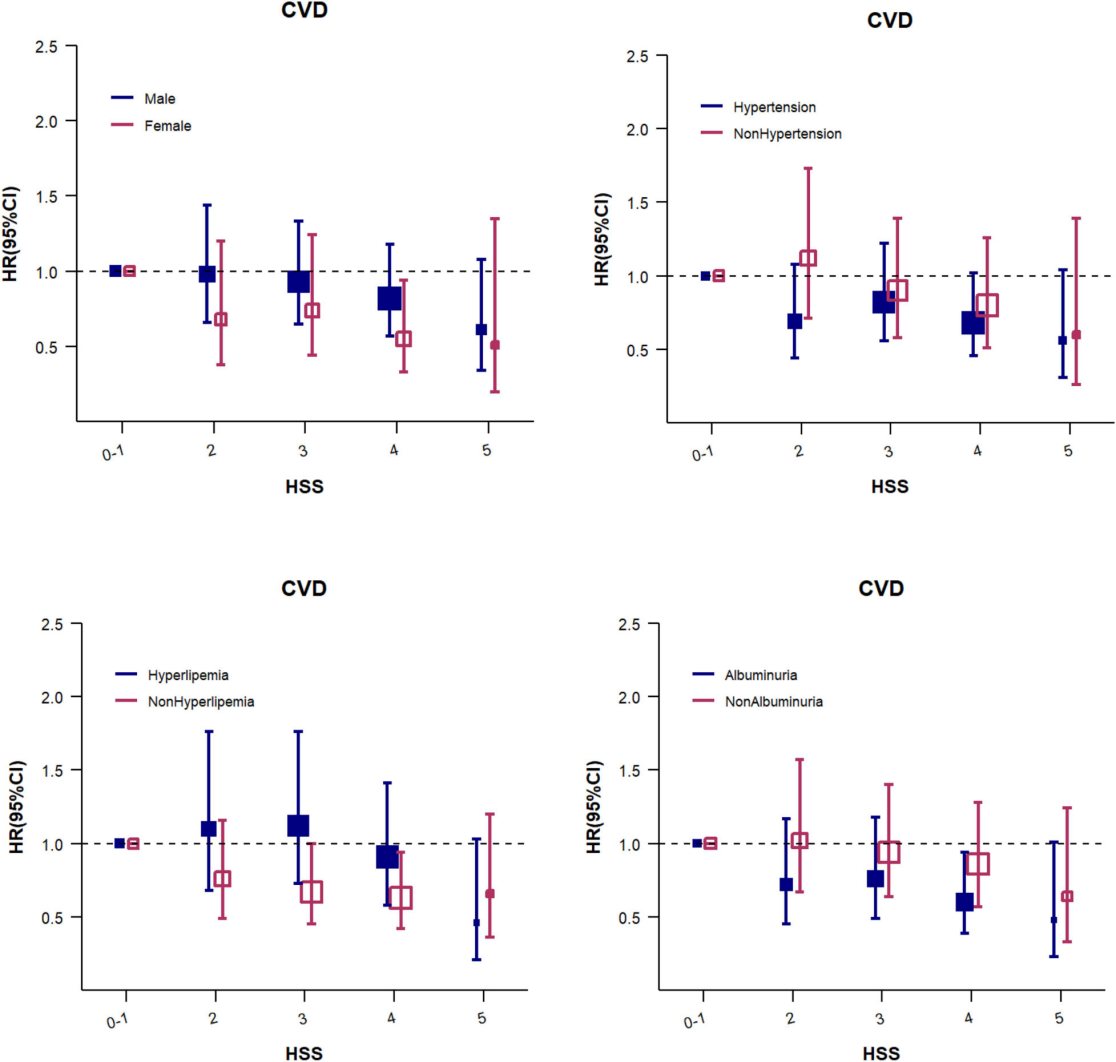
The HSS for CVD events (subgroup analysis). Adjusted for age, sex, education, heart rate, BMI, LDL-C, HDL-C, eGFR, uACR, HbA1c, hs-CRP, hypertension status, family history of CVD, smoking status, physical activity,and use of sleep-affecting medications. The size of the solid and hollow square markers is proportional to the sample size.

Considering the high prevalence of other metabolic diseases among diabetic patients, the patients were further evaluated in the subgroups of hypertension, hyperlipidemia, and albuminuria (all *P* for interaction >0.05). The results were consistent with those noted for the overall population (Figure 2; Supplementary Table S2).

### 3.6 Sensitivity analyses

In order to further validate the reliability of the above study results, sensitivity analyses were performed by sequentially excluding one sleep parameter at a time. The total score for the sleep parameters was 4 points, with the 0-point group serving as the reference for Cox regression analysis (Supplementary Table S2). The results were consistent with those observed for the overall population. Since the diagnosis of hypertension includes the use of antihypertensive medications, antihypertensive medication was not adjusted for in the main model. Next, the Cox regression analysis was repeated after excluding populations consuming antihypertensive, antidiabetic, lipid-lowering, and antiplatelet aggregation medications, and no significant changes were noted. After excluding participants who developed CVD within the first 2 years, the results were consistent with the primary findings (Supplementary Table S3). The competing risk analysis showed that compared to participants with scores of 0–1, those with scores of 4 and 5 had a 25% and 43% lower risk of CVD, respectively. When evaluated ordinarily, each additional HSS was associated with an 11% lower risk of CVD (HR for a one-point higher HSS = 0.89; 95% CI 0.83–0.96) (Supplementary Table S5).

## 4 Discussion

In this large prospective cohort study and community-based study, the association of five sleep behaviors, namely, the HSS integrating sleep duration, early sleep-wake patterns, insomnia, snoring, and daytime sleepiness, jointly with the incidence of CVD events was analyzed in a population with type 2 diabetes. It was confirmed that a higher HSS is associated with a lower risk of CVD. The results also revealed that the relationship between HSS and CVD was more evident in individuals with albuminuria.

Currently, most studies investigating the association between the combined score of 5 sleep factors and incident CVD risk focus on the general population (10–12). The SALT study (11) calculated the HSS based on sleep duration of 7–9 h/d, morning chronotype, insomnia symptoms, snoring, and daytime sleepiness, and demonstrated that a poor sleep pattern (HSS 0–1) was associated with a higher risk of CVDs compared to a healthy sleep pattern (HSS 4–5) (HR, 1.22; 95% CI, 1.05–1.41). In the UK Biobank study (10), where the ideal sleep duration was defined as 7–8 h/d, participants with a high HSS (4 or 5) had a 30% and 35% reduction in CVD risk, respectively, compared to those with a poor score (0–1). A stronger effect size was observed in another European study (12), where the 4-point and 5-point HSS groups had a 38% and 63% lower CVD risk, respectively, compared to a poor score (0–1), possibly due in part to the inclusion of apnoea (based on the Berlin questionnaire) in the HSS definition rather than snoring in the UK Biobank study. Despite variations in specific sleep constructs across these studies, such as snoring vs. apnoea, sleep duration cut-points, and the inclusion of chronotype, they all consistently demonstrate a dose-response relationship between healthier sleep patterns and reduced CVD risk.

Individuals with diabetes are not only at high risk for CVD but also have a high prevalence of sleep disorders. Our results confirmed that participants with a HSS of 4 or 5, compared with 0–1, had a 27%, and 42% lower risk of developing CVD in community population with type 2 diabetes, respectively. The risk of CVD decreased by 11% per one-point increment in the baseline HSS. Compared to the data from the general population, the results of this study indicate that having relatively healthy sleep patterns can also reduce the risk of CVD in individuals with diabetes. However, the degree of risk reduction is less significant than that in the general population. The results from two other studies on diabetic populations also suggest that a healthy sleep pattern can reduce CVD risk by 17%–31% (20, 21). A possible explanation is that individuals with diabetes may have more cardiovascular risk factors, such as hypertension and dyslipidemia. The protective effects of healthy sleep on the cardiovascular system might be somewhat offset by the presence of these additional risk factors. In this study, the HSS was associated with the risk of CVD events and cardiovascular disease, but its association with stroke was less clear. This aligns with the findings of most previous studies (22), and one potential reason is that stroke events are relatively infrequent in the studied population.

In this study, about 43.7% of individuals with type 2 diabetes had healthy sleep patterns (HSS scores of 4–5 points). In contrast, a study of the general population with five similar sleep factors reported that 58% of individuals exhibited healthy sleep patterns (10). This difference suggests that the overall sleep quality of individuals with type 2 diabetes may be poorer than that of the general population. In line with our study, several previous studies found that integrating sleep duration (23, 24) and insomnia (25) had higher risk of CVD.

The biological mechanisms through which multiple sleep patterns collectively affect CVD are complex, and no consensus has been reached on this matter. Existing evidence suggests that insomnia, often accompanied by sleep deprivation and poor sleep quality, can lead to insulin resistance (26), increased inflammatory mediator levels (27, 28), heightened sympathetic nervous system activity (29), and increased oxidative stress (30), and disrupts circadian rhythms (31), all of which promote the development of cardiovascular risk factors, such as diabetes, hypertension, obesity, and even atherosclerosis (32, 33). Excessive sleep duration may reduce the amount of time available for health-promoting behaviors, such as physical exercise (34, 35). Going to bed late can disrupt the body's natural circadian rhythm. Habitual snoring is often linked to sleep apnea, and louder or more frequent snoring tends to indicate more severe obstructive sleep apnea (OSA) (36). Excessive daytime sleepiness is a common sign of OSA. During sleep apnea episodes, repeated airway blockages cause drops in oxygen levels, spikes in blood pressure, carbon dioxide buildup, and frequent awakenings. These events activate the sympathetic nervous system and lead to changes in blood flow, increased oxidative stress, and chronic inflammation—factors that all contribute to the development and progression of cardiovascular disease (37, 38). In overweight and obese individuals, even without OSA, heavy snoring can thicken the walls of the carotid arteries and widen their diameter (39). Snoring can also raise triglyceride levels regardless of body weight (40). These various sleep problems often occur together and affect the heart and blood vessels through several overlapping pathways.

Considering that diabetes is often accompanied by other metabolic disorders, individuals with diabetes and metabolic syndrome are at an increased risk of CVD (41). In order to explore whether the presence of other metabolic diseases affects the relationship between sleep quality and CVD risk, a subgroup analysis was conducted in this study. The results revealed that, regardless of the presence of other metabolic diseases, there was a consistent trend of a decrease in CVD risk with increasing sleep score. The presence of hypertension, hyperlipidemia, or albuminuria did not significantly influence the strength or direction of the association between sleep score and the risk of CVD.

Considering that the mortality rate is higher in the diabetic population than in the general population, death may introduce a competing risk. Therefore, a competing risk analysis for death was conducted for the overall population. The results were consistent with those observed for the main model, which further ensures the reliability of the findings of this study. No studies, to date, have used competing risk models to analyze the impact of sleep scores on the incidence of new-onset CVD events in the diabetic population.

## 5 Limitations

Although this study provides additional evidence regarding the impact of sleep scores on the risk of CVD in the diabetic population, it has certain limitations. First, CVD events in this study were defined based on the disease diagnosis codes from hospital admission records, which may not have included patients with CVD who did not require hospitalization, although this group is probably quite small. Second, the sleep patterns were self-reported by participants, which may have led to misclassification, and “Early chronotype” was not included in this study, as a substitute, “early sleep-wake patterns” was used. Additionally, for ease of understanding and use by healthcare professionals and the general public, the sleep score was calculated by assigning equal weights to all indicators, which could have led to overestimating or underestimating the associations of certain indicators with CVD incidence. Furthermore, as an observational study, this research could not accurately establish the causal relationship between sleep scores and the development of CVD. Finally, the study population consisted primarily of male employees from the Kailuan Group; thus, the generalizability of the results may be limited. However, the sex-stratified results showed that the association direction between HSS and CVD risk in both male and female was consistent with that in the overall population, and there was no sex-based difference.

## 6 Conclusion

Achieving 4–5 optimal sleep patterns could prevent 30%–40% of CVD events in patients with type 2 diabetes, which emphasizes the strong impact of sleep on cardiovascular health in this population. Therefore, healthcare professionals and the general public should recognize that maintaining or improving sleep patterns is crucial for the prevention of CVD risk. The approach should be to control the traditional cardiovascular risk factors along with maintaining a good sleep pattern, as comprehensive management of multiple risk factors is necessary to significantly reduce the risk of CVD in patients with type 2 diabetes.

## Data Availability

The raw data supporting the conclusions of this article will be made available by the authors, without undue reservation.

## References

[1] Grandner MA Fernandez FX. The translational neuroscience of sleep: a contextual framework. Science. (2021) 374(6567):568–73. 10.1126/science.abj8188 34709899 PMC8761057

[2] Baranwal N Yu PK Siegel NS. Sleep physiology, pathophysiology, and sleep hygiene. Prog Cardiovasc Dis*.* (2023)77:59–69. 10.1016/j.pcad.2023.02.005 36841492

[3] Wang YX Zhang L Li CJ Qi X Fan YQ He JS Predicted 10-year cardiovascular disease risk and its association with sleep duration among adults in Beijing-Tianjin-Hebei region, China. Biomed Environ Sci. (2021) 34(10):803–13. 10.3967/bes2021.109 34782046

[4] Qin Y Liu R Wang Y Tang J Cong L Ren J Self-reported sleep characteristics associated with cardiovascular disease among older adults living in rural eastern China: a population-based study. Clin Interv Aging. (2022) 17:811–24. 10.2147/CIA.S361876 35611325 PMC9124474

[5] Huang T Mariani S Redline S. Sleep irregularity and risk of cardiovascular events: the multi-ethnic study of atherosclerosis. J Am Coll Cardiol. (2020) 75(9):991–9. 10.1016/j.jacc.2019.12.054 32138974 PMC7237955

[6] Grundy SM Stone NJ Bailey AL Beam C Birtcher KK Blumenthal RS 2018 AHA/ACC/AACVPR/AAPA/ABC/ACPM/ADA/AGS/APhA/ASPC/NLA/PCNA guideline on the management of blood cholesterol: a report of the American College of Cardiology/American Heart Association task force on clinical practice guidelines. Circulation. (2019) 139(25):e1082–143. 10.1161/CIR.0000000000000625 30586774 PMC7403606

[7] Shetty NS Parcha V Patel N Yadav I Basetty C Li C AHA life’s essential 8 and ideal cardiovascular health among young adults. Am J Prev Cardiol. (2022) 13:100452. 10.1016/j.ajpc.2022.100452 36636126 PMC9830108

[8] Lloyd-Jones DM Allen NB Anderson CAM Black T Brewer LC Foraker RE Life’s essential 8: updating and enhancing the American Heart Association’s construct of cardiovascular health: a presidential advisory from the American Heart Association. Circulation. (2022) 146(5):e18–43. 10.1161/CIR.0000000000001078 35766027 PMC10503546

[9] Nambiema A Lisan Q Vaucher J Perier MC Boutouyrie P Danchin N Healthy sleep score changes and incident cardiovascular disease in European prospective community-based cohorts. Eur Heart J. (2023) 44(47):4968–78. 10.1093/eurheartj/ehad657 37860848 PMC10719494

[10] Fan M Sun D Zhou T Heianza Y Lv J Li L Sleep patterns, genetic susceptibility, and incident cardiovascular disease: a prospective study of 385,292 UK biobank participants. Eur Heart J. (2020) 41(11):1182–9. 10.1093/eurheartj/ehz849 31848595 PMC7071844

[11] Wang Z Yang W Li X Qi X Pan KY Xu W. Association of sleep duration, napping, and sleep patterns with risk of cardiovascular diseases: a nationwide twin study. J Am Heart Assoc. (2022) 11(15):e025969. 10.1161/JAHA.122.025969 35881527 PMC9375484

[12] Zhong Q Qin Z Wang X Lan J Zhu T Xiao X Healthy sleep pattern reduce the risk of cardiovascular disease: a 10-year prospective cohort study. Sleep Med. (2023) 105:53–60. 10.1016/j.sleep.2023.03.003 36963321

[13] Bashier A Bin Hussain A Abdelgadir E Alawadi F Sabbour H Chilton R. Consensus recommendations for management of patients with type 2 diabetes mellitus and cardiovascular diseases. Diabetol Metab Syndr. (2019) 11:80. 10.1186/s13098-019-0476-0 31572499 PMC6761728

[14] Krueger PM Friedman EM. Sleep duration in the United States: a cross-sectional population-based study. Am J Epidemiol. (2009) 169(9):1052–63. 10.1093/aje/kwp023 19299406 PMC2727237

[15] Han H Wang Y Li T Feng C Kaliszewski C Su Y Sleep duration and risks of incident cardiovascular disease and mortality among people with type 2 diabetes. Diabetes Care. (2023) 46(1):101–10. 10.2337/dc22-1127 36383480

[16] American Diabetes Association. Standards of medical care in diabetes–2010. Diabetes Care. (2010) 33(Suppl 1):S11–61. 10.2337/dc10-S011 20042772 PMC2797382

[17] Wu S Huang Z Yang X Zhou Y Wang A Chen L Prevalence of ideal cardiovascular health and its relationship with the 4-year cardiovascular events in a northern Chinese industrial city. Circ Cardiovasc Qual Outcomes. (2012) 5(4):487–93. 10.1161/CIRCOUTCOMES.111.963694 22787064

[18] Inker LA Schmid CH Tighiouart H Eckfeldt JH Feldman HI Greene T Estimating glomerular filtration rate from serum creatinine and cystatin C. N Engl J Med. (2012) 367(1):20–9. 10.1056/NEJMoa1114248 22762315 PMC4398023

[19] Nowak C Ärnlöv J. Kidney disease biomarkers improve heart failure risk prediction in the general population. Circ Heart Fail. (2020) 13(8):e006904. 10.1161/CIRCHEARTFAILURE.120.006904 32757644

[20] Hu J Wang X Cheng L Dang K Ming Z Tao X Sleep patterns and risks of incident cardiovascular disease and mortality among people with type 2 diabetes: a prospective study of the UK biobank. Diabetol Metab Syndr. (2024) 16(1):15. 10.1186/s13098-024-01261-8 38212811 PMC10782582

[21] Yeap BB Marriott RJ Dwivedi G Adams RJ Antonio L Ballantyne CM Associations of testosterone and related hormones with all-cause and cardiovascular mortality and incident cardiovascular disease in men: individual participant data meta-analyses. Ann Intern Med. (2024) 177(6):768–81. 10.7326/M23-2781 38739921 PMC12768424

[22] Kadier K Qin L Ainiwaer A Rehemuding R Dilixiati D Du YY Association of sleep-related disorders with cardiovascular disease among adults in the United States: a cross-sectional study based on national health and nutrition examination survey 2005–2008. Front Cardiovasc Med. (2022) 9(4):954238. 10.3389/fcvm.2022.954238 35990939 PMC9386143

[23] Wang Y Huang W O'Neil A Lan Y Aune D Wang W Association between sleep duration and mortality risk among adults with type 2 diabetes: a prospective cohort study. Diabetologia. (2020) 63(11):2292–304. 10.1007/s00125-020-05214-4 32671413 PMC7527363

[24] Han H Cao Y Feng C Zheng Y Dhana K Zhu S Association of a healthy lifestyle with all-cause and cause-specific mortality among individuals with type 2 diabetes: a prospective study in UK biobank. Diabetes Care. (2022) 45(2):319–29. 10.2337/dc21-1512 34857534

[25] Hein M Lanquart JP Mungo A Loas G. Cardiovascular risk associated with co-morbid insomnia and sleep apnoea (COMISA) in type 2 diabetics. Sleep Sci. (2022) 15(Spec 1):184–94. 10.5935/1984-0063.20220018 35273765 PMC8889951

[26] Briançon-Marjollet A Weiszenstein M Henri M Thomas A Godin-Ribuot D Polak J. The impact of sleep disorders on glucose metabolism: endocrine and molecular mechanisms. Diabetol Metab Syndr. (2015) 7:25. 10.1186/s13098-015-0018-3 25834642 PMC4381534

[27] Yeghiazarians Y Jneid H Tietjens JR Redline S Brown DL El-Sherif N Obstructive sleep apnea and cardiovascular disease: a scientific statement from the American Heart Association. Circulation. (2021) 144(3):e56–67. 10.1161/CIR.0000000000000988 34148375

[28] Kadier K Dilixiati D Ainiwaer A Liu X Lu J Liu P Analysis of the relationship between sleep-related disorder and systemic immune-inflammation index in the US population. BMC Psychiatry. (2023) 23(1):773. 10.1186/s12888-023-05286-7 37872570 PMC10594811

[29] Labarca G Gower J Lamperti L Dreyse J Jorquera J. Chronic intermittent hypoxia in obstructive sleep apnea: a narrative review from pathophysiological pathways to a precision clinical approach. Sleep Breath. (2020) 24(2):751–60. 10.1007/s11325-019-01967-4 31758436

[30] Lavie L. Obstructive sleep apnoea syndrome–an oxidative stress disorder. Sleep Med Rev. (2003) 7:35–51. 10.1053/smrv.2002.0261 12586529

[31] Yin J Jin X Shan Z Li S Huang H Li P Relationship of sleep duration with all-cause mortality and cardiovascular events: a systematic review and dose-response meta-analysis of prospective cohort studies. J Am Heart Assoc. (2017) 6(9):e005947. 10.1161/JAHA.117.005947 28889101 PMC5634263

[32] St-Onge MP Grandner MA Brown D Conroy MB Jean-Louis G Coons M Sleep duration and quality: impact on lifestyle behaviors and cardiometabolic health: a scientific statement from the American Heart Association. Circulation. (2016) 134(18):e367–86. 10.1161/CIR.0000000000000444 27647451 PMC5567876

[33] Sarinc Ulasli S Sariaydin M Ozkececi G Gunay E Halici B Unlu M. Arterial stiffness in obstructive sleep apnoea: is there a difference between daytime and night-time?. Respirology. (2016) 21(8):1480–5. 10.1111/resp.12845 27381837

[34] Cassidy S Chau JY Catt M Bauman A Trenell MI. Cross-sectional study of diet, physical activity, television viewing and sleep duration in 233,110 adults from the UK biobank; the behavioural phenotype of cardiovascular disease and type 2 diabetes. BMJ Open. (2016) 6(3):e010038. 10.1136/bmjopen-2015-010038 27008686 PMC4800116

[35] Huang BH Duncan MJ Cistulli PA Nassar N Hamer M Stamatakis E. Sleep and physical activity in relation to all-cause, cardiovascular disease and cancer mortality risk. Br J Sports Med. (2022) 56(13):718–24. 10.1136/bjsports-2021-104046 34187783

[36] Kim JW Lee CH Rhee CS Mo JH. Relationship between snoring intensity and severity of obstructive sleep apnea. Clin Exp Otorhinolaryngol. (2015) 8(4):376–80. 10.3342/ceo.2015.8.4.376 26622957 PMC4661254

[37] Jia Y Liu C Li H Li X Wu J Zhao Y Enlarged perivascular space and its correlation with polysomnography indicators of obstructive sleep apnea. Nat Sci Sleep. (2021) 13:863–72. 10.2147/NSS.S305465 34211302 PMC8242141

[38] Maniaci A Iannella G Cocuzza S Vicini C Magliulo G Ferlito S Oxidative stress and inflammation biomarker expression in obstructive sleep apnea patients. J Clin Med. (2021) 10(2):277. 10.3390/jcm10020277 33451164 PMC7828672

[39] Taylor C Kline CE Rice TB Duan C Newman AB Barinas-Mitchell E. Snoring severity is associated with carotid vascular remodeling in young adults with overweight and obesity. Sleep Health. (2021) 7(2):161–7. 10.1016/j.sleh.2020.12.004 33402252 PMC8084936

[40] Hou FF Wang BB Chen Y Wang Q Wu Q Yan LN. Relationship between triglyceride levels and different snoring states: a systematic review and meta-analysis. Eur J Med Res. (2024) 29(1):641. 10.1186/s40001-024-02246-z 39741360 PMC11689565

[41] Alexander CM Landsman PB Teutsch SM Haffner SM. NCEP-defined metabolic syndrome, diabetes, and prevalence of coronary heart disease among NHANES III participants age 50 years and older. Diabetes. (2003) 52(5):1210–4. 10.2337/diabetes.52.5.1210 12716754

