# Supplementary material for: Correlation between healthy sleep score and risk of cardia-cerebrovascular disease among people with type 2 diabetes: a prospective cohort study
Source: Front Cardiovasc Med. 2026 Jan 8;12:1640125. doi: 10.3389/fcvm.2025.1640125 (PMC12823943; doi:10.3389/fcvm.2025.1640125)
Supplement: Supplementary file 1 [file Datasheet1.pdf]

Supplemental Online Content

Table S1. Multivariate Cox regression analysis of HSS effect on new-onset cardiovascular disease and stroke

Table S2. Multivariate Cox regression analysis of HSS effect on new-onset CVD events (Subgroup analysis)

Table S3. Multivariate Cox regression analysis of HSS effect on new-onset CVD events (Excluding one sleep factor at a time)

Table S4. Multivariate Cox regression analysis of HSS effect on new-onset CVD events (Sensitivity analysis)

Table S5. Multivariate Cox regression analysis of HSS effect on new-onset CVD events results(from Fine-Gray competitive risk models)

Table S1 Multivariate Cox regression analysis of HSS effect on new-onset cardiovascular disease and stroke

	cardiovascular disease			stroke		
	Case/No.	Median follow-up (years)	HR(95%CI)	Case/No.	Median follow-up (years)	HR(95%CI)
0-1	26/359	12.87	1	29/359	14.33	1
2	54/954	10.00	0.76(0.48, 1.22)	81/954	15.09	1.06(0.69, 1.62)
3	143/2268	11.25	0.82(0.54, 1.26)	175/2268	13.73	0.94(0.63, 1.39)
4	123/2536	8.76	0.64(0.42, 0.99)	169/2536	12.11	0.87(0.58, 1.30)
5	13/246	9.83	0.63(0.33, 1.20)	13/246	9.80	0.65(0.34, 1.25)
Per+1 score	359/6363	10.12	0.88(0.79, 0.99)	467/6363	13.19	0.92(0.83, 1.01)
P trend	-	-	0.032	-	-	0.085

Adjusted for age, sex, education, heart rate, BMI, LDL-C, HDL-C, eGFR, uACR, HbA1c, hs-CRP, hypertension status, family history of CVD, smoking status, physical activity, and use of sleep-affecting medications.

Table S2. Multivariate Cox regression analysis of HSS effect on new-onset CVD events (Subgroup analysis)

Outcome	No.	No./1000 PYs	0-1	2	3	4	5	P for interaction
Gender*								0.531
Male	162/1392	21.47	ref	0.98(0.67, 1.45)	0.93(0.65, 1.34)	0.83(0.57, 1.19)	0.61(0.34, 1.09)	
Female	628/4971	23.43	ref	0.68(0.38, 1.22)	0.75(0.45, 1.26)	0.56(0.33, 0.95)	0.51(0.33, 1.18)	
Hypertension#								0.461
No	333/3186	19.06	ref	1.13(0.71, 1.78)	0.90(0.58, 1.40)	0.81(0.52, 1.27)	0.60(0.26, 1.40)	
Yes	457/3177	27.07	ref	0.69(0.44, 1.08)	0.82(0.56, 1.22)	0.68(0.46, 1.02)	0.56(0.31, 1.02)	
Hyperlipemia								0.807
No	370/3206	21.12	ref	0.76(0.49, 1.14)	0.68(0.46, 1.01)	0.63(0.42, 0.95)	0.65(0.36, 1.22)	
Yes	420/3157	24.95	ref	1.10(0.69, 1.76)	1.12(0.72, 1.47)	0.91(0.58, 1.42)	0.46(0.22, 1.03)	
Albuminuria£								0.567
No	459/4214	19.68	ref	1.07(0.70, 1.64)	0.97(0.65, 1.38)	0.89(0.59, 1.34)	0.67(0.36, 1.29)	
Yes	331/2149	29.95	ref	0.69(0.43, 1.10)	0.74(0.48, 1.14)	0.58(0.38, 0.90)	0.47(0.23, 0.98)	

*Adjusted for age, education, heart rate, BMI, LDL-C, HDL-C, eGFR, uACR, HbA1c, hs-CRP, hypertension status, family history of CVD, smoking status, physical activity,and use of sleep-affecting medications. # Adjusted for age, sex, education, heart rate, BMI, LDL-C, HDL-C, eGFR, uACR, HbA1c, hs-CRP, family history of CVD, smoking status, physical activity,and use of sleep-affecting medications. £ Adjusted for age, sex, education, heart rate, BMI, LDL-C, HDL-C, eGFR, HbA1c, hs-CRP, family history of CVD, smoking status, physical activity, hypertension status, and use of sleep-affecting medications.

Table S3 Multivariate Cox regression analysis of HSS effect on new-onset CVD events (Excluding one sleep factor at a time)

	Excluded sleep duration	Excluded snoring	Excluded insomnia	Excluded daytime sleepiness	Excluded early Sleep-Wake Patterns
0	ref	ref	ref	ref	ref
1	1.03(0.42, 2.56)	0.85(0.60, 1.21)	0.91(0.44, 1.86)	0.76(0.42, 1.36)	0.75(0.30, 1.89)
2	0.79(0.32, 1.92)	0.82(0.60, 1.13)	0.86(0.43, 1.76)	0.73(0.41, 1.31)	0.74(0.31, 1.82)
3	0.74(0.31, 1.79)	0.73(0.53, 1.01)	0.78(0.38, 1.48)	0.63(0.35, 1.13)	0.63(0.26, 1.54)
4	0.59(0.23, 1.51)	0.63(0.39, 1.00)	0.55(0.25, 1.23)	0.46(0.23, 0.94)	0.56(0.23, 1.55)
Per+1 score	0.87(0.79, 0.96)	0.91(0.84, 0.98)	0.89(0.82, 0.98)	0.88(0.81, 0.96)	0.88(0.82, 0.95)
P trend	0.006	0.015	0.014	0.004	0.002

Adjusted for age, sex, education, heart rate, BMI, LDL-C, HDL-C, eGFR, uACR, HbA1c, hs-CRP, hypertension status, family history of CVD, smoking status, physical activity,and use of sleep-affecting medications.

Table S4 Multivariate Cox regression analysis of HSS effect on new-onset CVD events (Sensitivity analysis)

	Excluded diabetes medication n=3227	Excluded antihypertensive medication n=5272	Excluded lipid lowering medication n=6287	Excluded antiplatelet medication n=6272	Excluded events within the first 2 years n=6058
0-1	ref	ref	ref	ref	
2	1.06(0.60, 1.90)	0.94(0.64, 1.38)	0.92(0.66, 1.27)	0.87(0.62, 1.21)	0.84(0.57, 1.24)
3	0.99(0.58, 1.71)	0.88(0.61, 1.25)	0.87(0.64, 1.17)	0.85(0.63, 1.15)	0.91(0.64, 1.30)
4	0.82(0.48, 1.41)	0.78(0.54, 1.12)	0.75(0.56, 1.03)	0.73(0.53, 0.99)	0.76(0.53, 1.09)
5	0.59(0.26, 1.35)	0.69(0.39, 1.21)	0.59(0.36, 0.97)	0.59(0.36, 0.97)	0.52(0.28, 0.95)
Per+1 score	0.88(0.79, 0.99)	0.91(0.83, 0.99)	0.90(0.83, 0.97)	0.90(0.83, 0.97)	0.91(0.83, 0.99)
P trend	0.043	0.043	0.006	0.006	0.031

Adjusted for age, sex, education, heart rate, BMI, LDL-C, HDL-C, eGFR, uACR, HbA1c, hs-CRP, hypertension status, family history of CVD, smoking status, physical activity,and use of sleep-affecting medications.

Table S5. Multivariate Cox regression analysis of HSS effect on new-onset CVD events results(from Fine-Gray competitive risk models)

	Death competing risk analysis<br>n=6135
HSS	
0-1	ref
2	0.91(0.66, 1.25)
3	0.88(0.66, 1.19)
4	0.75(0.55, 1.00)
5	0.57(0.35, 0.93)
Per+1 score	0.89(0.83, 0.96)
P trend	0.002

Adjusted for age, sex, education, heart rate, BMI, LDL-C, HDL-C, eGFR, uACR, HbA1c, hs-CRP, hypertension status, family history of CVD, smoking status, physical activity and use of sleep-affecting medications. The model satisfies the proportional hazards assumption (p = 0.335).
